# Exploring the causal role and mechanism of galanin in glioblastoma: integration of mendelian randomization, network analysis, molecular docking and experimental validation

**DOI:** 10.3389/fphar.2026.1916071

**Published:** 2026-08-04

**Authors:** Haimin Song, Yuxuan Jia, Xiaofen Qiu, Tao Long, Wenfei Yang

**Affiliations:** 1 Department of Neurosurgery, First Affiliated Hospital of Gannan Medical University, Ganzhou, Jiangxi, China; 2 The First Clinical Medical College of Gannan Medical University, Ganzhou, Jiangxi, China; 3 Central Laboratory, Ganzhou People’s Hospital, Ganzhou, Jiangxi, China; 4 Department of Otolaryngology Head and Neck Surgery, First Affiliated Hospital of Gannan Medical University, Ganzhou, Jiangxi, China

**Keywords:** galanin, glioblastoma, *in vitro* experiments, mendelian randomization, molecular docking, network analysis

## Abstract

**Background:**

Glioblastoma (GBM) is a highly malignant central nervous system tumor with an exceptionally poor prognosis. The neuropeptide Galanin is implicated in GBM development, but its specific molecular mechanisms and therapeutic potential remain unclear. In this study, we integrate Mendelian randomization (MR) and network toxicology approaches to systematically characterize the pharmacological activity and mechanistic underpinnings of Galanin in GBM.

**Methods:**

A two-sample MR analysis was employed to investigate the causal relationship between Galanin and GBM risk. Galanin-related targets and differentially expressed genes were screened from public databases. Prognostic genes were identified through protein-protein interaction networks, single-cell sequencing, and the LASSO-COX model, and binding activity was validated by molecular docking. Finally, target genes were silenced using siRNA, and qRT-PCR, CCK-8, and colony formation assays were performed to validate the regulatory roles of CCNA2 and SERPINA1 in GBM cell proliferation.

**Results:**

The MR analysis confirmed a causal association between Galanin and GBM risk. Multidimensional bioinformatics identified four core genes: CCNA2, BRCA1, SERPINA1, and LGALS3. Molecular docking confirmed stable binding of Galanin to the proteins encoded by these genes. *In vitro* experiments demonstrated that silencing CCNA2 or SERPINA1 effectively inhibits GBM cell proliferation and colony formation.

**Conclusion:**

This study provides a comprehensive analysis of the regulatory mechanisms of Galanin in GBM from both genetic and computational toxicology perspectives. Our findings indicate that Galanin modulates CCNA2, BRCA1, SERPINA1, and LGALS3, likely through mediating the PI3K-Akt and focal adhesion signaling pathways, thereby hindering tumor progression. These results provide a foundational rationale for Galanin-targeted therapeutic strategies in GBM. Furthermore, the *in vitro* experiments corroborate that CCNA2 and SERPINA1 promote tumor cell proliferation, positioning them as promising therapeutic targets for GBM.

## 1 Introduction

The incidence rate of primary malignant brain tumors is approximately 7 per 100,000 people, with glioblastoma accounting for about 49% of all brain tumors (Schaff and Mellinghoff, 2023). Therefore, glioblastoma is the most common malignant primary brain tumor in adults, with its incidence increasing after age 40 and peaking among adults aged 75 to 84 (Wen et al., 2020). The age-adjusted incidence rate of glioblastoma in the United States is 3.22 per 100,000 people, and it increases with older age at diagnosis and in males; incidence rates also vary across the globe. Glioblastoma contributes disproportionately to morbidity and mortality, with an overall 5-year relative survival rate of only 6.8% (Vargas López, 2021). Although our understanding of glioma biology has deepened in recent years, the prognosis for patients with glioblastoma remains poor, with IDH wild-type glioblastoma patients having a median overall survival of 12–21 months and only about 7% surviving 5 years. Patients with IDH-mutant tumors lacking 1p/19q co-deletion have a median overall survival of 7–8 years, while those with 1p/19q co-deleted oligodendroglial achieve a median overall survival of 13–14 years following chemoradiotherapy (Bell et al., 2020). Under the standard treatment regimen of surgery, radiation therapy, and temozolomide (TMZ) chemotherapy, the median overall survival (OS) for rigorously selected patients in clinical trials is approximately 15–18 months, while the 5-year survival rate is less than 10%. Once glioblastoma recurs, the median overall survival is estimated to be 24–44 weeks (Vargas López, 2021). This grim prognostic outlook places a heavy burden on modern healthcare systems— —not only due to the substantial loss of life-years resulting from high mortality rates, but also because of the high direct and indirect economic costs associated with long-term neurological deficits, cognitive impairment, and care needs. Coupled with the limited efficacy of existing treatments and the scarcity of options following recurrence, GBM has become one of the most challenging diseases in the field of oncology, urgently requiring more in-depth mechanistic research and innovative treatment strategies (Poniatowski et al., 2024).

Galanin is a neuropeptide initially isolated from the porcine intestine, composed of 29–30 amino acids (Tatemoto et al., 1983). It is widely expressed in the nervous and enteroendocrine systems of mammals, where it colocalizes with various neurotransmitters and inhibits synaptic transmission (Cheung et al., 1985; Mazarati et al., 2000). Physiologically, it participates in numerous neuroendocrine regulatory processes, including pain perception, sleep, cognition, energy metabolism, and reproduction; in the spinal cord, it regulates pain perception through multiple mechanisms and impairs learning and memory abilities (Kerr et al., 2000; Hökfelt and Tatemoto, 2008). Galanin expression is upregulated in pathological states; mutations in the human GAL gene can induce dominant-inherited temporal lobe epilepsy accompanied by hearing impairment (Guipponi et al., 2015). Additionally, this neuropeptide is involved in metabolic regulation, including insulin metabolism and intestinal smooth muscle activity (Gesmundo et al., 2017; Autio et al., 2020). Previous studies have confirmed that GAL-LI and GAL binding sites are distributed throughout the central nervous system and its tumors, and that GAL and its receptors are present in glial cells (Berger et al., 2003). However, the mechanisms by which it regulates glioblastoma remain unclear. This study integrates network toxicology, machine learning, molecular docking techniques, and *in vitro* experiments to systematically investigate the mechanisms by which Galanin influences glioblastoma progression.

Network toxicology integrates bioinformatics with multi-omics technologies to construct association networks of compounds, targets, and signaling pathways based on databases (Huang, 2023; Cheng et al., 2025). This technology can elucidate complex multi-component and multi-target interactions and perform visual analysis, providing a research framework for protein interaction analysis and the prediction of toxicological mechanisms (Hoeng et al., 2014). Molecular docking technology simulates the binding patterns of ligands and target proteins at the atomic level to predict their interaction mechanisms and biological effects. In the field of toxicology, the combination of these two technologies can clarify the interactions between harmful substances and biomolecules, revealing their toxicological mechanisms and potential risks of harm to the human body (He et al., 2024).

## 2 Methods

### 2.1 Causal effect analysis of galanin and GBM

This study employed Mendelian randomization (MR) analysis to investigate the causal relationship between Galanin and glioblastoma (GBM), strictly adhering to the three core assumptions of MR (Zeng et al., 2023; Zeng et al., 2025). Using the HG19/GRCh37 reference genome, we retrieved Galanin-related GWAS data from the IEU database (21,758 European participants); simultaneously, we extracted aggregated GBM GWAS data from the Finngen R10 database, which included 253 cases and 314,193 controls.

The selection criteria for instrumental variables were as follows: significant genetic loci were identified using a p-value threshold of p < 5 × 10^−8^ (Zhang et al., 2022); linkage disequilibrium (LD) SNPs were filtered based on the 1000 Genomes European Reference Panel, with *r* ^2^ < 0.001 and a linkage interval of 10,000 kb, while SNPs associated with confounding factors were excluded (Auton et al., 2015); this study employed inverse-variance weighting (IVW) as the primary analysis model, utilizing the R package TwoSampleMR (version 0.6.20) to conduct the analysis, in combination with multiple auxiliary models; the robustness of the results was assessed using the MR-Egger intercept test and the horizontal pleiotropy test (Bowden et al., 2015).

### 2.2 Acquire galanin targets

The SMILES for Galanin was retrieved from the PubChem (https://pubchem.ncbi.nlm.nih.gov/) database by searching for “Galanin” (Kim et al., 2025). Using the SEA database (https://sea.bkslab.org/), relevant targets for Galanin were identified via its isomeric SMILES (Xie et al., 2025). The Galanin SDF molecular format was converted to mol2 format using ChemBio3D software, and corresponding targets were identified through the PharmMapper database (https://lilab-ecust.cn/pharmmapper/index.html) (Wang et al., 2017). The species selected for analysis was “*Homo sapiens*,” with the following selection criteria: PharmMapper z'-score >0 (Shi et al., 2020). All targets were annotated using the UniProt knowledge base search function within the Protein Database (UniProt Consortium, 2025) (https://www.uniprot.org/) to assign their official symbols (2025). Galanin targets obtained from multiple databases were consolidated to derive a comprehensive target set for Galanin, facilitating subsequent research.

### 2.3 Collection of GBM-Related targets

We obtained relevant data (GSE86574 and GSE147352) by accessing the Gene Expression Omnibus (GEO) using GEOquery in R software. Differentially expressed genes (DEGs) were identified for GSE86574 and GSE147352 using the limma and DESeq2 R packages, respectively, with default parameter settings (adjusted P-value <0.05, |log2FoldChange| > 1) (Anders and Huber, 2010).

### 2.4 Protein-protein interaction network construction process and hub target screening strategy

Using a bioinformatics platform (https://bioinfogp.cnb.csic.es/tools/venny/), we generated a Venn diagram to identify overlapping targets between Galanin and GBM. Subsequently, we performed protein-protein interaction (PPI) analysis on these targets using the STRING database (https://cn.string-db.org/), restricting species to *H. sapiens* and setting the minimum interaction score threshold to medium confidence (>0.4). The STRING analysis results were imported into Cytoscape version 3.10.2 for PPI network visualization and node parameter calculation (Liu et al., 2024). Using the cytoHubba plugin within the software, the top 30 key targets were identified based on metrics including Degree, Betweenness Centrality, and Closeness Centrality. Subsequently, a Venn diagram was employed to filter and refine the hub targets. Finally, the MCODE plugin in Cytoscape was utilized to validate the hub genes obtained from the PPI network clustering analysis.

### 2.5 Analysis of target gene function and pathway enrichment

This study employed R language to conduct bioinformatics analysis on the 76 intersecting genes identified through screening. Primary R packages utilized included ClusterProfiler, org. Hs.e.g.,.db and ggplot2. ClusterProfiler was specifically applied to Gene Ontology (GO) enrichment analysis and Kyoto Encyclopedia of Genes and Genomes (KEGG) pathway analysis, while also enabling visualization of results (Yu et al., 2012).

To ensure the reliability and statistical significance of the results, strict filtering criteria were established: only enrichment entries with p-values <0.05 and corrected p-values <0.05 were retained to control for errors arising from multiple hypothesis testing. Based on these criteria, statistically significant enriched GO entries and KEGG pathways were ultimately identified, closely related to the functions of the intersecting genes.

### 2.6 Analysis of hub genes in glioblastoma based on single-cell RNA sequencing

This study analyzed single-cell RNA sequencing data using the Seurat package to investigate the expression profiles of hub genes. Data were sourced from the GSE131928 and CGGA datasets in the Gene Expression Omnibus (GEO) database (Neftel et al., 2019; Zhao et al., 2021). Following the operating guidelines provided by 10× Genomics, a digital gene expression matrix was constructed from the raw data using the CellRanger 2.2.0 analysis workflow. Subsequently, R software was used to filter, normalize, and cluster the raw digital gene expression matrix (i.e., the UMI counts for each gene in each cell). The filtering criteria for cells and genes are as follows: to exclude multicellular bodies, the total number of UMIs per detected cell must be less than (1 – the binomial rate), where the binomial rate is determined according to the 10× Genomics user manual; to exclude environmental background noise, the UMI count must be above the 8th percentile; to exclude dead cells, the proportion of mitochondrial RNA must be below the 90th percentile. Analysis of single-cell transcriptomics data for all samples was performed in accordance with the manuals for the Seurat and ClusterProfiler software packages (Ni et al., 2023). We focused on analyzing the expression levels of hub genes across different cell subpopulations and visualized the results using methods such as dot plots.

### 2.7 Model construction

To construct a robust prognostic feature model for glioblastoma and effectively prevent overfitting, this study employed the LASSO-Cox regression method to systematically analyze transcriptomic sequencing data from the TCGA and CGGA databases (Zheng et al., 2024). Through this algorithm, hub genes associated with Galanin were identified, forming the basis for establishing a prognostic model termed the “GBM-Galanin Feature.” During model construction, we utilized the TCGA glioblastoma dataset (691 samples) and randomly divided it into a training set and an internal validation set at a 7:3 ratio. An independent CGGA glioblastoma dataset (970 samples) served as the external validation set to comprehensively evaluate the model’s generalization capability and reliability.

### 2.8 Investigating the interaction between hub genes and galanin via molecular docking

This study obtained the two-dimensional structure of Galanin (in SDF format) from the PubChem database and minimized its energy using ChemBio 3D software to derive its three-dimensional conformation. The receptor protein structure was sourced from high-resolution human protein crystal structures archived in the RCSB Protein Data Bank (https://www.rcsb.org/). Prior to docking, proteins underwent preprocessing in PyMOL 2.5.0, including water molecule removal, hydrogenation, and charge calculation (Song et al., 2024). Subsequently, molecular docking was performed using the HDOCK server (http://hdock.phys.hust.edu.cn/): this platform first conducted a global conformation search via Fast Fourier Transform (FFT), followed by iterative optimization of binding modes based on a knowledge-based scoring function (Yan et al., 2020). The complex conformation with the optimal Docking Score and Confidence Score was ultimately selected, and results were visualized using PyMOL (Liu and Pu, 2025).

### 2.9 Cell siRNA transfection

siRNA oligonucleotides were synthesized by Gene Pharma (Shanghai, China). The sequence of si-Negative Ctrl: sense 5′-3’: UUCUCCGAACGUGUCACGUTT; antisense 5′-3’: ACGUGA CACGUUCGGAGAATT. The sequence of si-CCNA2 #1: sense 5′-3’: GUAGCAGAGUUUGUGUACATT; antisense 5′-3’: UGU ACACAAACUCUGCUACTT. The sequence of si-CCNA2 #2: sense 5′-3’: GGGAGAAUUAAGUUUGAUATT; antisense 5′-3’: UAUCAAACUUAAUUCUCCCTT. The sequence of si-CCNA2 #3: sense 5′-3’: GGAUCUUCCUGUAAAUGAUTT; antisense 5′- 3’: AUCAUUUACAGGAAGAUCCTT. The sequence of si- SERPINA1 #1: sense 5′-3’: GAGGCCAAGAAACAGAUCATT; antisense 5′-3’: UGAUCUGUUUCUUGGCCUCTT. The sequence of si-SERPINA1 #2: sense 5′-3’: GCCCAGAAGACA GAUACAUTT; antisense 5′-3’: AUGUAUCUGUCUUCUGGG CTT. The sequence of si-SERPINA1 #3: sense 5′-3’: GCCUGA AGCUAGUGGAUAATT; antisense 5′-3’: UUAUCCACUAGC UUCAGGCTT. Lipofectamine™ RNAiMAX transfection reagent (Invitrogen, 13,778,150) was used for siRNA transfection according to the standard procedure.

### 2.10 Experiments on cell proliferation-related functions

Total RNA was extracted from siRNA-treated U251 and U87 cells using TRIzol reagent. After verifying purity and concentration, cDNA was synthesized via reverse transcription, and qRT-PCR experiments were conducted. The PCR reaction protocol included a pre-denaturation step followed by 40 amplification cycles. Using GAPDH as the internal control, the relative expression levels of the CCNA2 and SERPINA1 genes were calculated using the 2^−ΔΔCq^ method to evaluate gene silencing efficiency. All experiments were performed in triplicate (Wen et al., 2025). The relevant primer sequences are detailed in Table 1. Among the three tested siRNAs, the two with the highest and most reproducible silencing efficiencies for each gene were selected for subsequent functional assays.

**TABLE 1:** Primer sequences and product lengths of genes.

Species	Genes	Sequences	Amplification product length (bp)
Homo	SERPINA1	FORWARD:ACTGTCAACTTCGGGGACAC	162
		REVERSE:GGGTCTCTCCCATTTGCCTT	
Homo	CCNA2	FORWARD:CTGGTGGTCTGTGTTCTGTGA	144
		REVERSE:TCTTGGATGCCAGTCTTACTCA	

Transfected glioblastoma cells were seeded into 96-well plates, and absorbance at 450 nm was measured at 0, 24, 48, and 72 h. Proliferation curves were plotted to analyze differences in cell proliferation activity. Additionally, cells in the logarithmic growth phase were seeded into 6-well plates and cultured at a constant temperature for 10 days. After washing with PBS, paraformaldehyde fixation, and crystal violet staining, long-term proliferation and colony-forming ability were assessed based on colony morphology and number.

## 3 Results

### 3.1 Summary of this study

**FIGURE 1:**
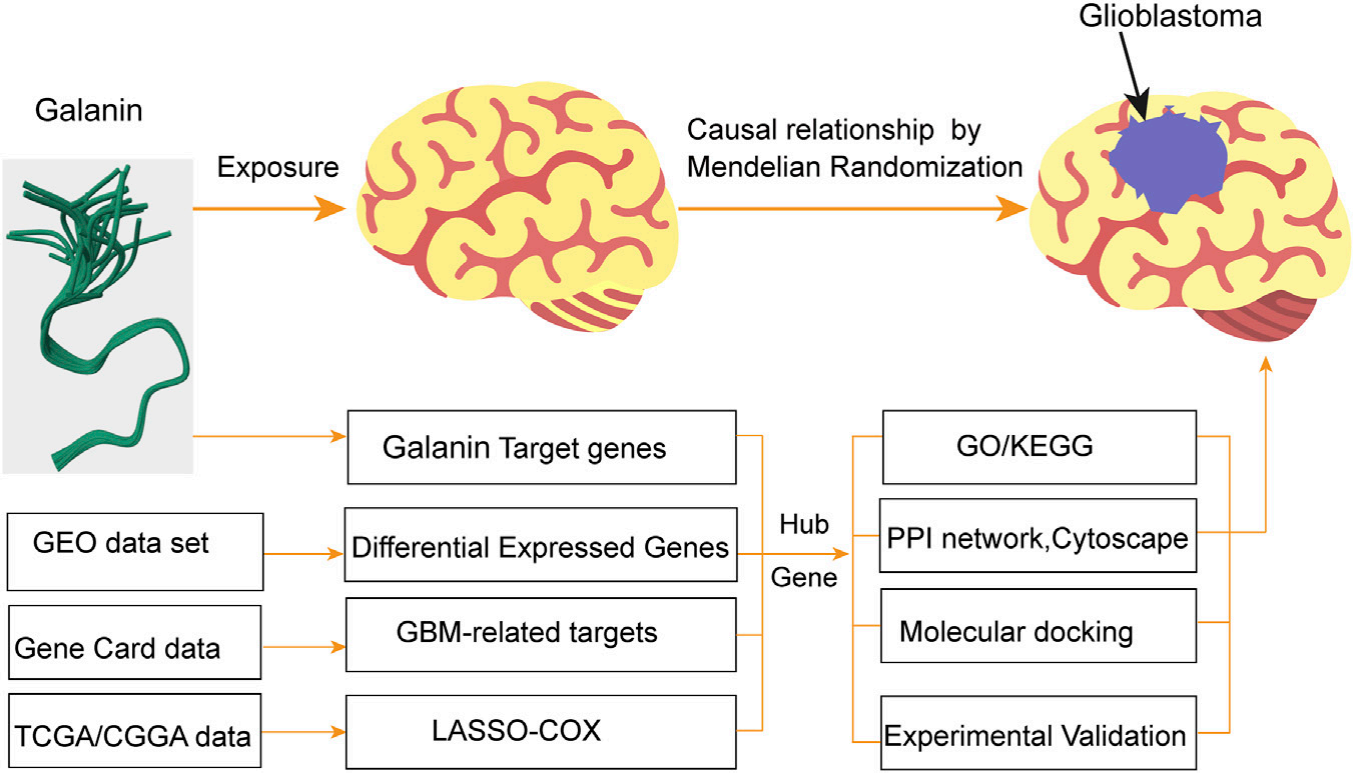
Research and analysis process diagram.

Figure 1 illustrates the overall workflow of this study. First, using Galanin as the exposure and glioblastoma (GBM) as the outcome, we investigated the causal relationship between the two using Mendelian randomization analysis. Concurrently, we identified Galanin’s target genes and screened for differentially expressed genes in GBM from the GEO database; we also collected GBM-related targets by integrating the GeneCard database with the TCGA and CGGA databases. After taking the intersection of the aforementioned targets, we constructed a protein-protein interaction (PPI) network and performed topological analysis using Cytoscape to identify hub genes. Subsequently, GO functional annotation and KEGG pathway enrichment analysis were performed on the hub genes. Furthermore, based on TCGA and CGGA data, a prognostic model was constructed using LASSO-Cox regression, and the binding affinities between key molecules and targets were predicted via molecular docking. Finally, functional validation of the core targets was conducted through experimental verification.

### 3.2 Mendelian randomization analysis suggests a causal association between galanin and glioblastoma risk

**FIGURE 2:**
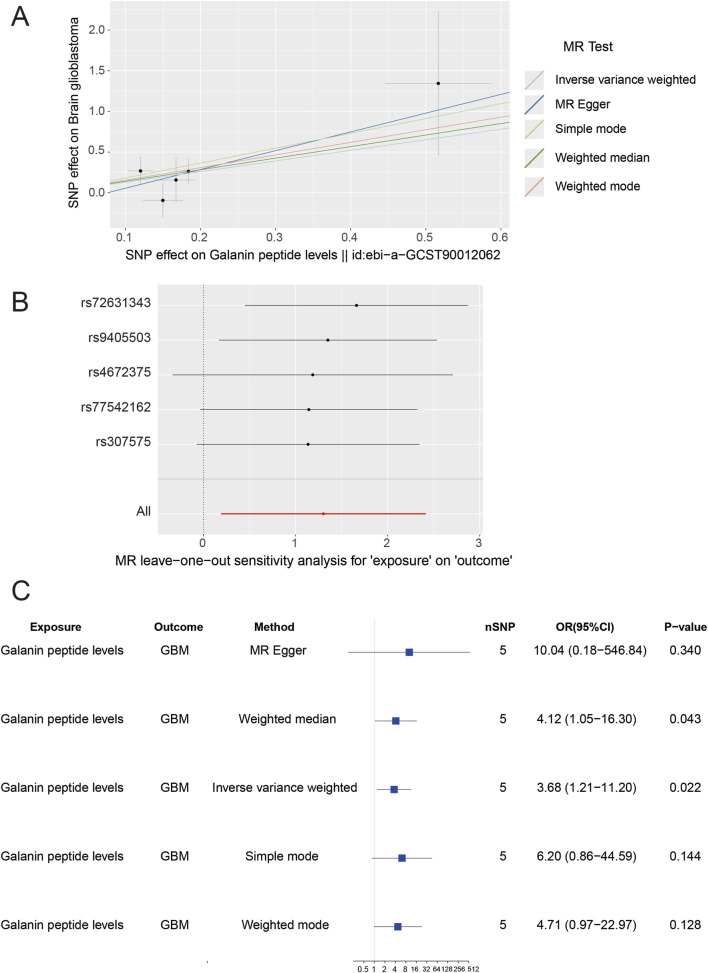
The impact of Galanin peptide levels on GBM using MR analysis. **(A)** The scatter plot of 5 SNPs applied 5 MR test methods; **(B)** the leave-one-out sensitivity analysis plot for “Galanin” on “GBM”; **(C)** Forest plot showing the OR value and P value of the effect of exposure on the outcome.

We performed a two-sample Mendelian randomization (MR) analysis using R software, with genetic variants associated with Galanin as the exposure and glioblastoma genetic data as the outcome. Five single-nucleotide polymorphisms (SNPs) were initially selected as instrumental variables at a genome-wide significance threshold of P < 5 × 10^−8^. After excluding SNPs with potential confounding effects or in linkage disequilibrium, five independent valid instruments were retained. To assess the strength of the selected instrumental variables, we calculated the F-statistics for each SNP and used F > 10 as an empirical threshold for excluding bias from weak instrumental variables. The calculated F-values for all SNPs are detailed in Supplementary Table S1. The inverse-variance weighting (IVW) method revealed a significant causal association between Galanin and glioblastoma (P < 0.05), and the scatter plot confirmed a positive correlation (Figure 2A). Leave-one-out sensitivity analysis confirmed the robustness of the findings, demonstrating that they were not driven by any single instrumental variable (Figure 2B). The MR effect estimate was positive (IVW odds ratio [OR] = 3.68, 95% confidence interval [CI]: 1.21–11.20, P = 0.022), confirming that Galanin promotes glioblastoma development (Figure 2C). Both Cochran’s Q test and the test for horizontal pleiotropy yielded non-significant results, indicating no obvious heterogeneity or pleiotropy in the analysis (Supplementary Table S2). Overall, genetic evidence indicates a causal association between Galanin and glioblastoma risk.

### 3.3 Identifying galanin-GBM-associated hub genes by integrating target prediction and transcriptomics data

**FIGURE 3:**
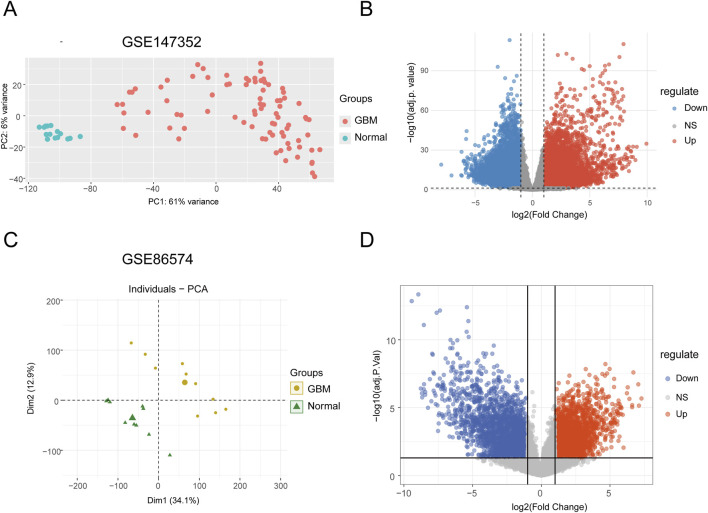
DEGs of the GBM obtained from GEO database. **(A)** GSE147352 Principal Component Analysis plot, distribution of DEGs in normal and tumor groups; **(B)** Volcano Plot of DEGs in GSE147352, the red color represented upregulated genes, the blue represented downregulated genes and the grey represented normal genes; **(C)** GSE86574 Principal Component Analysis plot, distribution of DEGs in normal and tumor groups; **(D)** Volcano Plot of DEGs in GSE86574, the red color represented upregulated genes, the blue represented upregulated genes and the grey represented normal genes.

**FIGURE 4:**
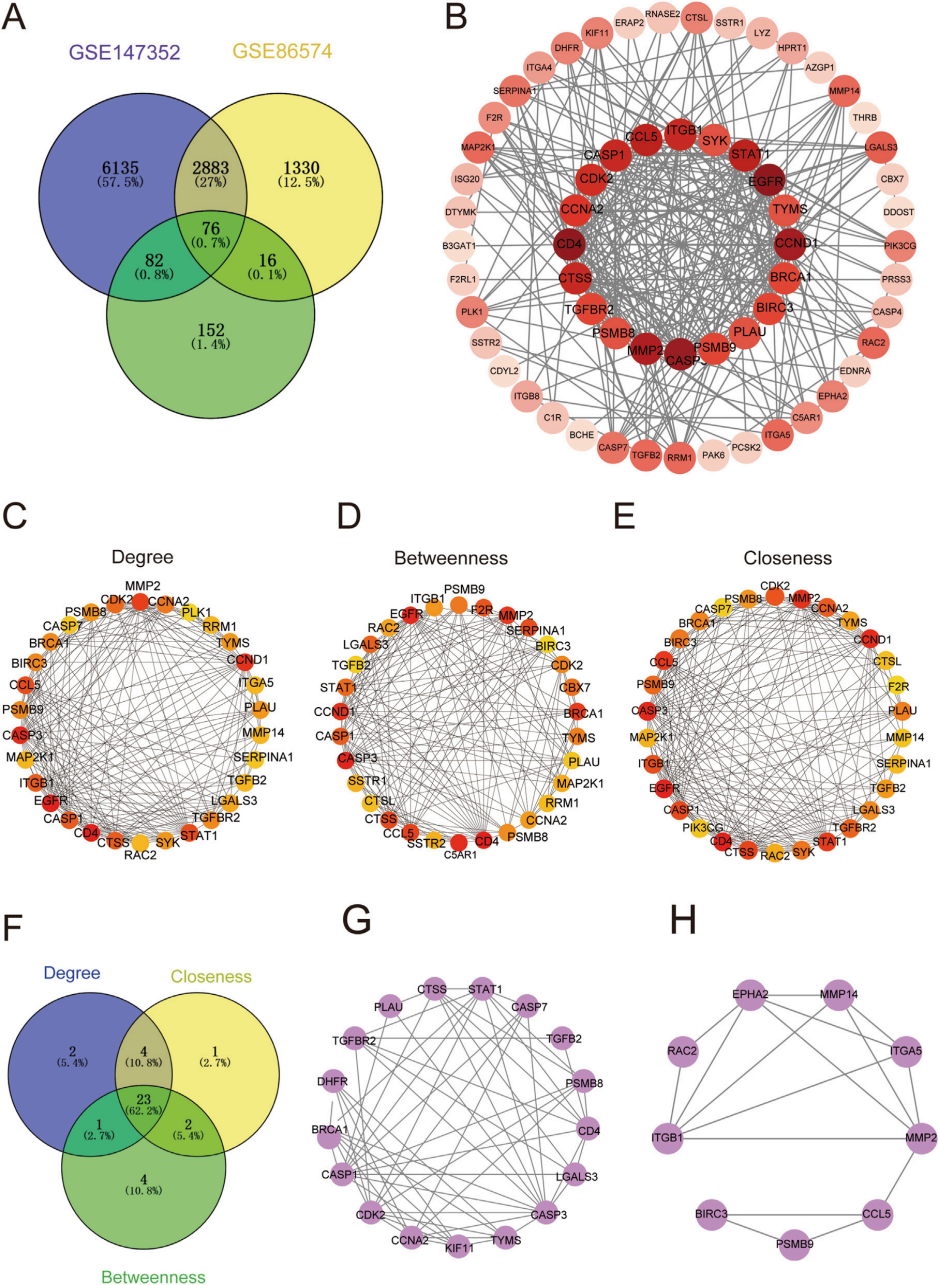
Venn and network diagrams of intersection genes and hub genes screening respectively. **(A)** Venn diagram of common genes; **(B)** the PPI network of intersection genes and core targets network. **(C)** Degree analysis results of top 30; **(D)** Betweenness Centrality analysis results of top30. **(E)** Closeness Centrality analysis results of top 30; **(F)** Venn diagram of hub genes; **(G)** the first MCODE plug-in analysis results; **(H)** the second MCODE plug-in analysis results.

This study integrated data from the SEA and PharmMapper databases to preliminarily identify 326 Galanin-related targets. Differential expression analyses between glioblastoma (GBM) and normal brain tissues were subsequently performed using the limma and DESeq2 packages on the GSE147352 and GSE86574 datasets, respectively. Principal component analysis (PCA) revealed that the primary principal components effectively distinguished the two sample groups in both datasets, whereas the subsequent components accounted for relatively limited sample variance. Applying the screening criteria of adjusted P < 0.05 and |log_2_FC| > 1, we identified 9,176 and 4,305 differentially expressed genes (DEGs) from the two datasets, respectively (Figures 3B,D; Supplementary Table S3). Finally, intersecting these DEGs with the Galanin-predicted targets yielded a total of 76 overlapping genes (Figure 4A; Supplementary Table S3, S4). These were used to construct a potential functional network, serving as core targets for investigating the pharmacological mechanisms by which Galanin regulates glioblastoma.

### 3.4 Construction of the PPI network and analysis of hub genes

In this study, we constructed a protein-protein interaction (PPI) network based on the STRING database using 76 overlapping genes and performed visualization analysis using Cytoscape v3.10.0 (Figure 4B). The network comprises 61 nodes and 285 interaction edges. Using the cytoHubba plugin, the top 30 genes were selected based on three topological features: degree, betweenness centrality, and closeness centrality. The intersection of these sets yielded the core hub genes (Figures 4C–F); details of the relevant genes are provided in Supplementary Table S5. Furthermore, the MCODE plugin was used to perform subnetwork clustering analysis on the PPI network, identifying two highly correlated functional modules (Figures 4G,H). In summary, this analytical framework identified key genes and functional clusters, providing a theoretical basis for elucidating the molecular mechanisms by which Galanin regulates GBM.

### 3.5 KEGG and GO enrichment analysis results for intersecting genes

**FIGURE 5:**
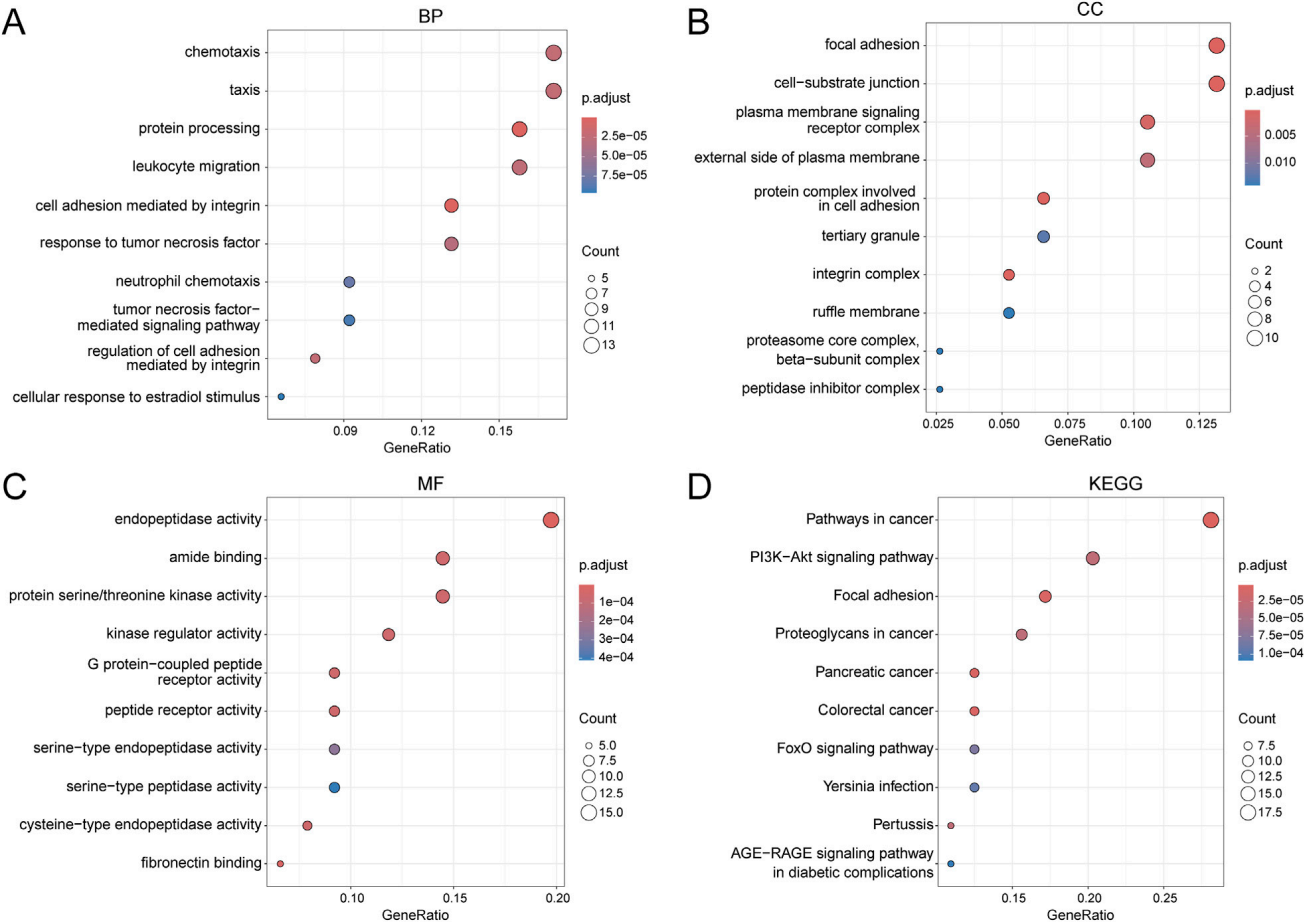
GO and KEGG Enrichment Analysis. Vertical axis indicates enrichment items, The horizontal axis is the Gene ratio, which represents the ratio of the number of genes of the item to all genes. **(A)** Biological processes (BP) analysis; **(B)** cellular components (CC) analysis; **(C)** molecular functions (MF) analysis; **(D)** KEGG enrichment analysis.

Through Gene Ontology (GO) analysis of 76 shared genes, we obtained several key findings: First, the intersecting genes were found to participate in several key biological processes (BP) depicted in Figure 5A, including chemotaxis, taxis, protein processing, leukocyte migration, cell adhesion mediated by integrin, response to tumor necrosis factor, neutrophil chemotaxis, tumor necrosis factor-mediated signaling pathway, regulation of cell adhesion mediated by integrin, and cellular response to estradiol stimulus. Second, cellular components (CC): Intersecting genes primarily localize within multiple cellular components, including focal adhesion, cell-substrate junction, plasma membrane signaling receptor complex, external side of plasma membrane, protein complex involved in cell adhesion, tertiary granule, integrin complex, ruffle membrane, proteasome core complex, beta-subunit complex, and peptidase inhibitor complex (Figure 5B). Third, co-expressed genes exhibit diverse molecular function (MF) profiles. These functions encompass endopeptidase activity, amide binding, protein serine/threonine kinase activity, kinase regulator activity, G protein-coupled peptide receptor activity, peptide receptor activity, serine-type endopeptidase activity, serine-type peptidase activity, cysteine-type endopeptidase activity, and fibronectin binding (Figure 5C). KEGG pathway enrichment analysis (Figure 5D) indicates that Galanin may influence glioblastoma development by regulating multiple key signaling pathways. Specifically, these pathways include Pathways in cancer, PI3K-Akt signaling pathway, Focal adhesion, Proteoglycans in cancer, Pancreatic cancer, Colorectal cancer, FoxO signaling pathway, *Yersinia* infection, Pertussis, and AGE-RAGE signaling pathway in diabetic complications. This analysis suggests that biological processes regulated by Galanin intervention are closely associated with malignant GBM phenotypes such as the tumor microenvironment and cellular invasion, providing theoretical support for its potential clinical translational value.

### 3.6 Results of model construction

**FIGURE 6:**
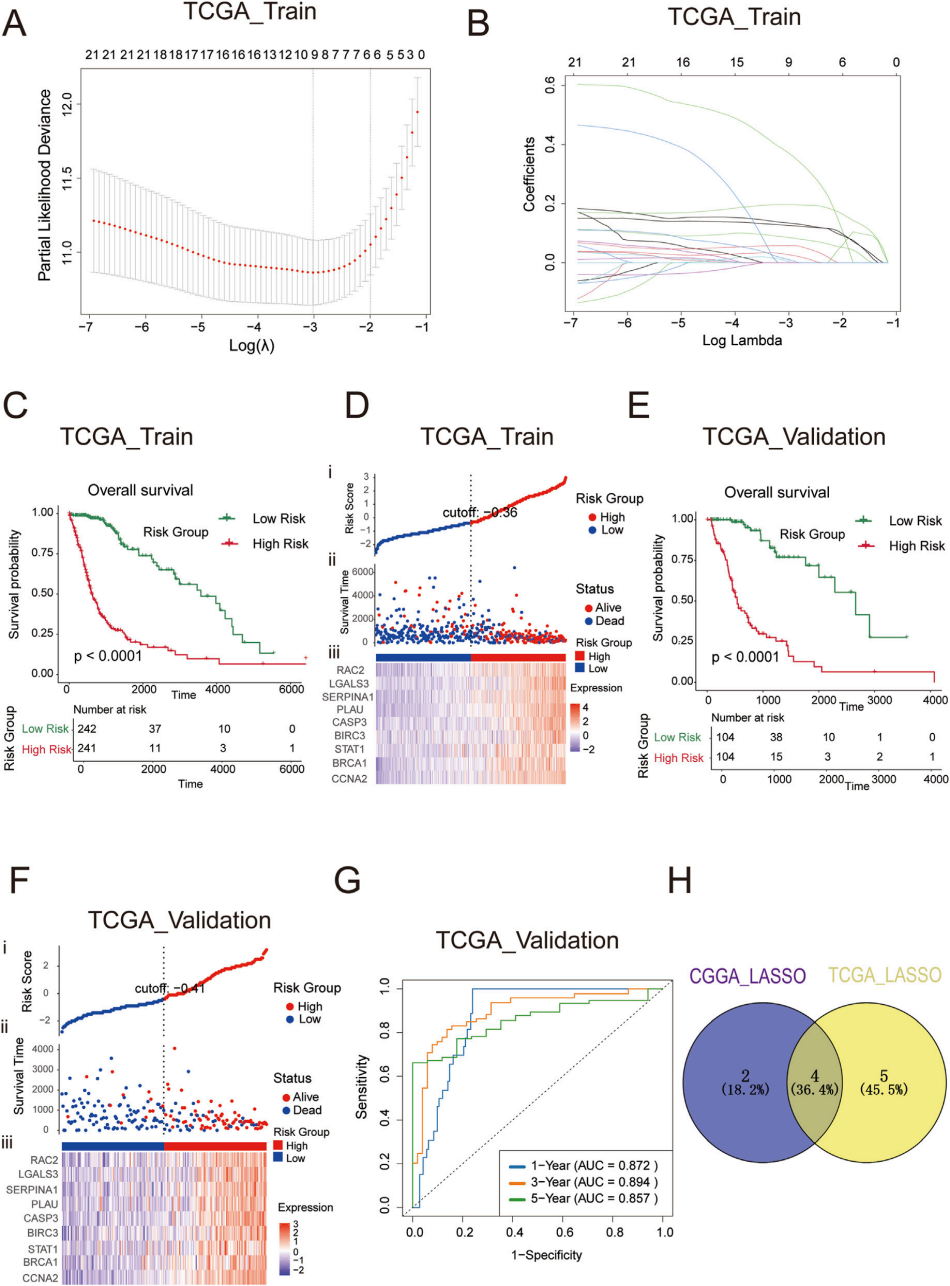
LASSO-COX analysis of 23 hub genes in TCGA glioblastoma datasets. **(A,B)** LASSO-COX analysis in the training set. **(C)** Survival analysis of high-vs. low-risk patients in the training set. **(D)** Coefficient paths (i), risk score distribution (ii), and survival status scatter plot (iii) from the training set; the optimal lambda (lambda.1se) selected 9 genes. **(E)** Kaplan-Meier survival analysis in the TCGA_Validation set. **(F)** Risk scores, survival status, and expression profiles of the 9 genes in the validation set. **(G)** ROC curves with AUC values in the validation set. **(H)** Intersection of hub genes identified by TCGA_LASSO and CGGA_LASSO.

To identify key prognostic genes associated with Galanin and construct a robust prognostic model, this study employed LASSO-Cox regression analysis based on TCGA glioblastoma transcriptome data. Through this method, we developed the “GBM-Galanin signature” prognostic model. Specifically, the TCGA dataset (691 samples) was randomly divided into a training set and an internal validation set at a 7:3 ratio for model development and preliminary performance validation, respectively. At the optimal λ value, nine genes with non-zero coefficients were selected (RAC2, LGALS3, SERPINA1, PLAU, CASP3, BIRC3, STAT1, BRCA1, CCNA2), forming the final model (Figures 6A,B). The fitted risk score formula is: Risk Score = 0.13444 × ExpCCNA2 + 0.3732 × ExpBRCA1 + 0.00456 × ExpSTAT1 + 0.02745 × ExpBIRC3 + 0.12589 × ExpPLAU +0.05787 × ExpSERPINA1 + 0.19668 × ExpLGALS3 + 0.01114 × ExpRAC2, we calculated each patient’s risk score and categorized them into high-risk and low-risk groups based on the median score. Figure 6D illustrates the risk score distribution, patient survival time, survival status, and expression patterns of these nine feature genes. Notably, all protective genes exhibit high expression in the low-risk group. Kaplan-Meier survival analysis in the TCGA training set demonstrates significantly better prognosis for low-risk patients compared to high-risk patients (P < 0.0001, Figure 6C). To assess model robustness, the same formula was applied to the internal validation set TCGA_Validation (Figure 6E). Results confirmed that high-risk groups remained significantly associated with poor prognosis in the validation cohort. Risk score distribution, survival status, and partial gene expression profiles (Figure 6F) in this validation set were consistent with the training set. Further ROC curve analysis assessed the model’s predictive performance for 1-year, 3-year, and 5-year survival rates in the TCGA_Validation cohort, yielding AUC values of 0.872, 0.894, and 0.857, respectively (Figure 6G), indicating robust predictive accuracy.

To validate the robustness of the Galanin-associated prognostic genes and feature model in independent data, we replicated the LASSO-Cox regression analysis workflow on the CGGA glioblastoma transcriptome dataset (970 samples). This dataset was similarly randomly split into training (70%) and validation (30%) sets. At the optimal λ value, six genes with non-zero coefficients were identified (LGALS3, SERPINA1, CDK2, TGFB2, BRCA1, CCNA2) (Supplementary Figures S1A,B), yielding the corresponding risk score formula (Risk Score = 0.26857 × ExpCCNA2 + 0.19059 × ExpBRCA1 + 0.12311 × ExpTGFB2 + 0.20396 × ExpCDK2 + 0.10872 × ExpSERPINA1 + 0.07923 × ExpLGALS3). After stratifying high-risk and low-risk groups based on median risk scores, Supplementary Figure S1D illustrates the risk scores, survival times, survival statuses, and expression distributions of these six genes, revealing that protective genes are highly expressed in the low-risk group. Kaplan-Meier analysis of the CGGA training set confirmed significantly superior overall survival in the low-risk group compared to the high-risk group (P < 0.0001, Supplementary Figure S1C). Applying the same scoring formula to the CGGA validation cohort (Supplementary Figure S1E), the high-risk group remained significantly associated with poor prognosis, with consistent risk score distribution, survival status, and gene expression patterns (Supplementary Figure S1F) compared to the training cohort. ROC curve analysis revealed AUC values of 0.751, 0.809, and 0.832 for predicting 1-year, 3-year, and 5-year survival rates in the CGGA validation cohort (Supplementary Figure S1G).

These findings were consistently validated across two independent datasets (TCGA and CGGA), collectively demonstrating that the constructed GBM-Galanin prognostic signature possesses robust discriminatory capability and predictive value across different cohorts. Based on the validation of their predictive efficacy, these four hub genes serve as potential focal points for Galanin action, and we will conduct more in-depth research on them.

### 3.7 Single-cell RNA sequencing analysis

**FIGURE 7:**
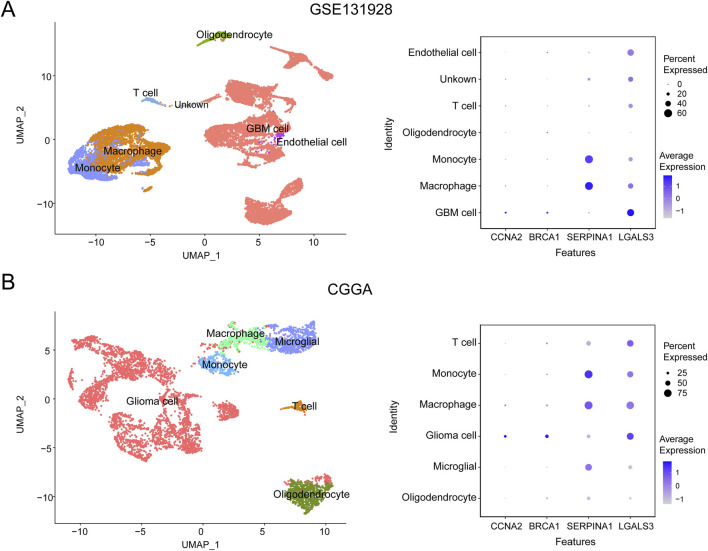
Results of single cell sequencing analysis. **(A)** UMAP (left) and dot plots (right) were produced based on cell grouping of single cell RNA data from GSE131928; **(B)** cell grouping of single cell RNA data from CGGA. The size of the dots represents the proportion of cells with high expression of a specific gene in the cell type, and the color intensity represents the average expression level in the expressed cells in bins.

This study analyzed single-cell RNA sequencing data from clinical glioblastoma samples to elucidate the expression profiles of key genes. The complete list of marker genes is provided in Supplementary Table S6. Cell clustering and annotation results revealed that genes such as CCNA2, BRCA1, SERPINA1, and LGALS3 were significantly expressed in both tumor cells and various immune cells; among these, SERPINA1 and LGALS3 were widely distributed across various cell populations (Figure 7). The results suggest that, in addition to acting on glial cells, Galanin may also participate in GBM progression by regulating the tumor immune microenvironment. The relevant key genes mediate interactions between tumor cells and the immune microenvironment, providing a basis for investigating its immune regulatory functions.

### 3.8 Molecular docking results between hub genes and galanin

**FIGURE 8:**
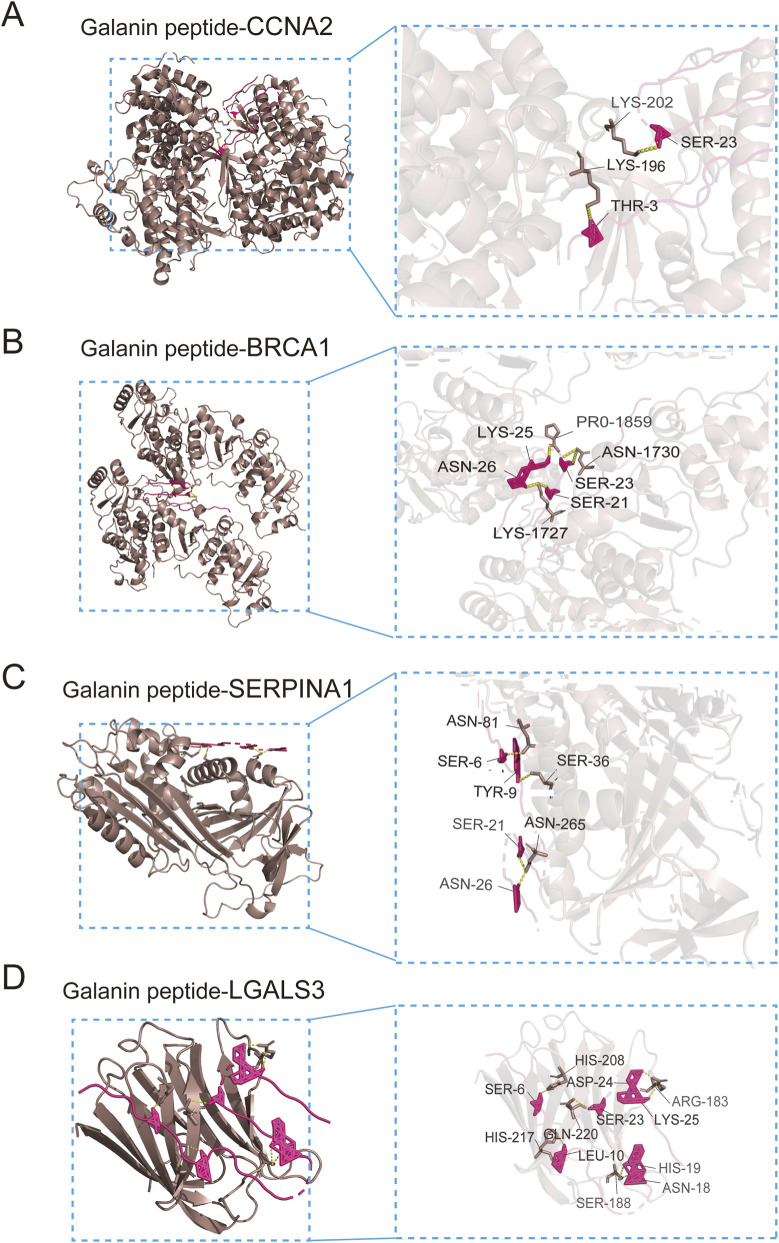
Optimal docking results for CCNA2, BRCA1, SERPINA1, and LGALS3. **(A)** Galamin peptide-CCNA2; **(B)** Galamin peptide-BRCA1; **(C)** Galamin peptide-SERPINA1; **(D)** Galamin peptide-LGALS3. Receptor proteins are depicted in light purple, ligand proteins in rose red, and hydrogen bonds are indicated by yellow dashed lines.

Molecular docking was further employed to characterize the interactions between Galanin and four core proteins, namely, CCNA2, BRCA1, SERPINA1, and LGALS3. Using the HDOCK platform, we successfully simulated docking poses for Galanin with each target protein. Notably, all four proteins exhibited high binding affinity to Galanin, as reflected by low docking scores (Supplementary Table S7). These findings not only demonstrate that Galanin can spontaneously form stable complexes with these key targets but also reinforce its important role in GBM pathogenesis. As shown in Figure 8, we generated three-dimensional structural models of the Galanin–target complexes, which clearly display the lowest-energy binding conformations and reveal potential hydrogen-bonding interactions. The docking models provide evidence for binding feasibility and offer detailed interaction insights, facilitating a structural-level understanding of how Galanin may regulate the functions of these pivotal proteins.

### 3.9 Reduced levels of CCNA2 and SERPINA1 inhibit glioblastoma cell proliferation

Previous studies have shown that abnormal BRCA1 expression in glioblastoma is associated with poor patient prognosis and increased tumor invasiveness (Harbi and Aschner, 2024); LGALS3 is a key regulatory molecule that mediates the malignant progression of glioblastoma and contributes to the establishment of a tumor-suppressive immune microenvironment (Zhao et al., 2024). Given that the biological functions of these two genes have been well-documented, this study selected the remaining two candidate genes identified through bioinformatics screening to conduct *in vitro* functional experiments, further investigating their roles and regulatory mechanisms in the development and progression of glioblastoma.

**FIGURE 9:**
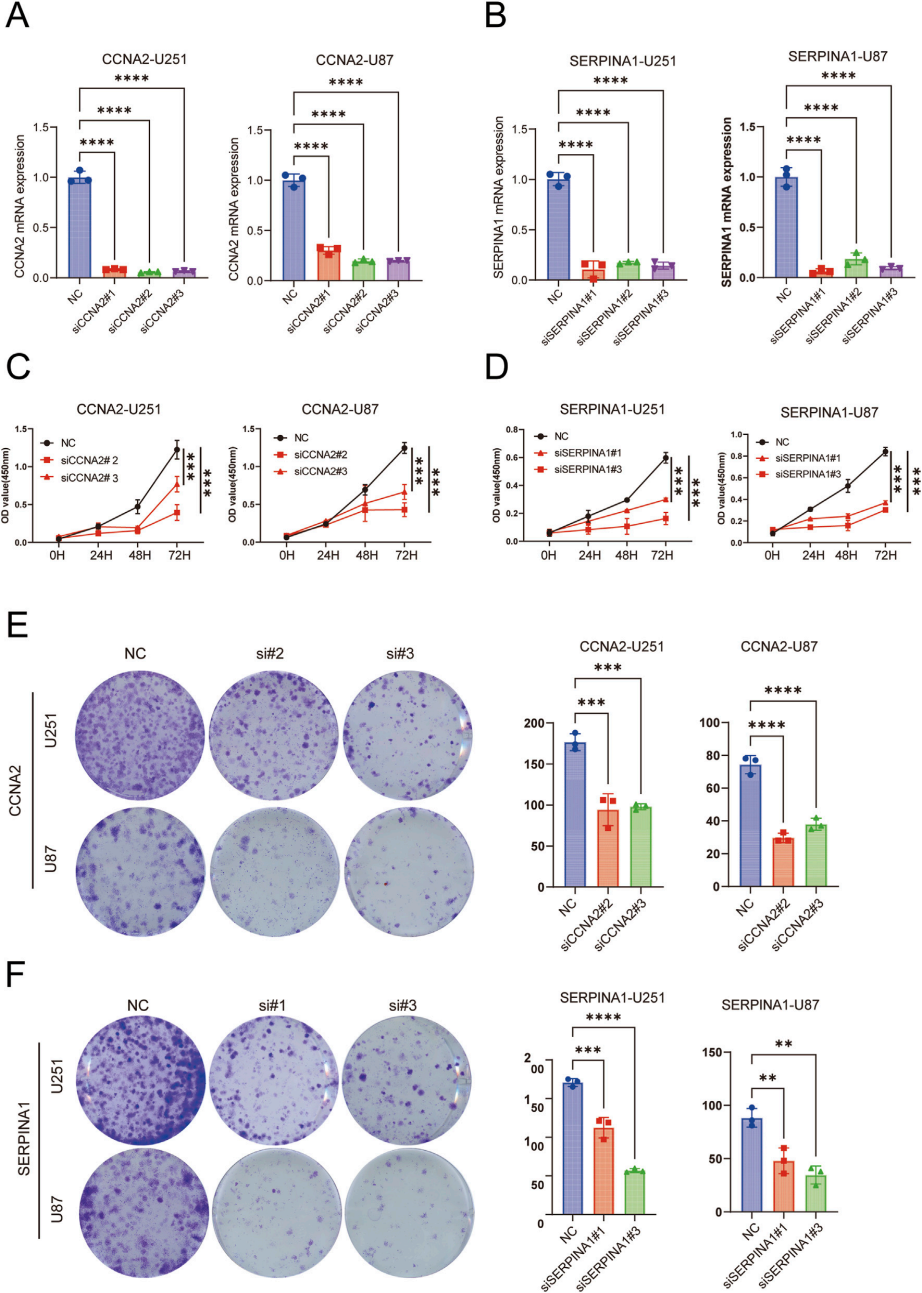
**(A,B)** qRT-PCR was used to detect the mRNA expression levels of the CCNA2 and SERPINA1 genes in U251 and U87 cells, respectively, to verify the efficiency of siRNA knockdown; **(C,D)** Changes in the proliferation activity of glioblastoma cells following knockdown of CCNA2 and SERPINA1 were assessed using the CCK-8 assay; **(E,F)** The ability of cells to form colonies following knockdown of CCNA2 and SERPINA1 was evaluated using a colony formation assay.

To investigate the roles of CCNA2 and SERPINA1 in glioblastoma cell proliferation, we first used siRNA technology to knock down these two genes in U251 and U87 cells, respectively, and validated the knockdown efficiency via qRT-PCR. The results showed that, compared with the negative control (NC) group, siRNA targeting CCNA2 (siCCNA2#1/#2/#3) significantly reduced the expression levels of CCNA2 mRNA in both cell lines (Figure 9A, p < 0.0001); similarly, siRNA targeting SERPINA1 (siSERPINA1#1/#2/#3) effectively knocked down SERPINA1 mRNA expression (Figure 9B, p < 0.0001), indicating that the experimental conditions were valid for subsequent functional assays.

Subsequently, we performed CCK-8 assays to assess the impact of gene knockdown on cell proliferation. The results showed that in both U251 and U87 cells, knockdown of CCNA2 (siCCNA2#2/#3 groups) significantly reduced the proliferation rate compared to the NC group, and the inhibitory effect on proliferation gradually increased with prolonged culture time (0–72 h) (Figure 9C). Consistent with this, knockdown of SERPINA1 (siSERPINA1#1/#3 group) similarly significantly inhibited the proliferation of both cell types, with markedly flattened growth curves (Figure 9D).

To further validate the effects of gene knockdown on long-term cell proliferation and clonogenic capacity, we performed clonogenic assays. The results showed that in U251 and U87 cells, the NC group formed numerous dense clonal colonies; whereas after knockdown of CCNA2 (si#2/#3 group) or SERPINA1 (si#1/#3 group), the cells’ clonogenic capacity significantly decreased, with a marked reduction in the number of clones and a decrease in their size (Figures 9E,F). These results suggest that knockdown of either CCNA2 or SERPINA1 significantly inhibits both the short-term proliferative activity and long-term clonogenic capacity of glioblastoma cells, indicating that both play a key role in the regulation of glioblastoma cell proliferation.

## 4 Discussion

This study integrates omics, machine learning, and computational biology approaches to investigate the biological role of Galanin in glioblastoma. A two-sample Mendelian randomization analysis has, for the first time, demonstrated from a genetic perspective that Galanin is positively associated with the risk of GBM, suggesting that this neuropeptide may play a carcinogenic role. Previous studies have only reported an association between the two but have not yet elucidated the specific mechanisms involved (Ohtaki et al., 1999; Santic et al., 2006). By utilizing MR analysis to account for confounding variables and reverse causality bias, this study provides high-quality evidence for the role of Galanin in modulating GBM and opens up a new avenue for targeted therapeutic strategies. The F-statistics confirmed that our instrumental variables were sufficiently robust to exclude weak instrument bias, reinforcing the reliability of the causal estimate. To validate our instrumental variables, we evaluated biological evidence linking each SNP to Galanin. Strong support was noted for rs4672375 (a cis-pQTL within GAL) and rs307575 (significantly associated with circulating Galanin) (Folkersen et al., 2017; Beck et al., 2023). For rs9405503 and rs72631343, we infer potential regulatory effects via linkage disequilibrium or distal enhancer-related eQTL mechanisms, though direct validation is lacking for the latter. Of note, rs9405503 has been identified as a genome-wide significant variant in a GWAS on circulating proteins (GCST90012062), supporting its potential relevance to galanin-related pathways. We acknowledge that rs77542162, located in ABCA6, lacks evidence for direct Galanin modulation. Nevertheless, leave-one-out analyses confirmed that excluding this SNP did not materially alter the causal estimates, supporting the robustness of our findings. Future functional studies, including fine-mapping and allelic expression assays, are warranted to definitively elucidate the regulatory architecture of these less-characterized variants. Overall, while two of the five SNPs lack direct mechanistic evidence, the collective strength of the instruments, combined with rigorous sensitivity checks, supports the reliability of our MR findings.

Next, through protein–protein interaction network analysis and LASSO-Cox regression, we identified four core hub genes: CCNA2, BRCA1, SERPINA1, and LGALS3. We acknowledge that, as a neuropeptide, Galanin presents inherent limitations when analyzed using tools such as PharmMapper and SEA, which are primarily optimized for small-molecule compounds. To mitigate potential algorithmic bias, we implemented a stringent screening threshold (z'-score >0) and cross-validated the predicted targets against GBM-differentially expressed genes as well as prognosis-associated genes. Furthermore, we conducted *in vitro* cellular experiments to functionally validate the two most promising core targets (CCNA2 and SERPINA1).

Cyclin A2 is encoded by the CCNA2 gene located on chromosome 4 at q27. It binds to and activates CDK1/CDK2, thereby regulating the G1/S and G2/M phase transitions of the cell cycle (Pagano et al., 1992). Current research has demonstrated that CCNA2 is abnormally expressed in various types of malignant tumors, can promote the development of glioblastoma, and can also regulate the epithelial-mesenchymal transition process in various cancer cells (Qi et al., 2020; Wang et al., 2021; Zhou et al., 2024).

The tumor suppressor gene BRCA1 is located at 17q21 and plays a key role in biological processes such as DNA damage repair and cell survival; it is widely expressed in proliferating tissues in adults, and deletion of this gene can trigger apoptosis (Xu et al., 1999; Zheng et al., 2000; Navaraj et al., 2009). Currently, there is no consensus on the role of BRCA1 in glioblastoma; the impact of the DNA damage repair pathways it mediates on the tumor changes as the cancer progresses and is closely associated with tumor resistance (Halazonetis et al., 2008; Squatrito et al., 2010).

Serpin peptidase inhibitor clade A member 1 (SERPINA1) is primarily synthesized in the liver and encodes α1-antitrypsin (AAT), a glycoprotein that inhibits various proteases, including neutrophil elastase and trypsin (Winkler et al., 2005; Pastor et al., 2006). Previous studies have demonstrated that SERPINA1 is abnormally overexpressed in breast cancer, colorectal cancer, and glioblastoma (Kwon et al., 2015; Yang et al., 2015). Its high expression in high-grade glioblastomas correlates with poor patient prognosis (Ookawa et al., 2018).

The LGALS3 gene encodes galectin-3, a galectin that recognizes and binds to β-galactosylated sugars on the surface of target cells (Trimboli et al., 2017; Bouffette et al., 2023). Galectin-3 is widely distributed in the cytoplasm, nucleus, and extracellular matrix, and is involved in biological processes such as cell proliferation, adhesion, angiogenesis, and apoptosis (Orlandi et al., 1998; Dong et al., 2018). LGALS3 is abnormally expressed in multiple tumors including hepatocellular carcinoma, prostate cancer, and other cancers (Knapp et al., 2013; Min et al., 2013). In glioblastoma, this gene is also closely associated with tumor prognosis and treatment resistance (Wang et al., 2019).

This study identified the pathways involved in Galanin-mediated regulation of glioblastoma through KEGG and GO enrichment analyses. The PI3K-Akt and focal adhesion pathways mediate tumor cell biological activities and malignant invasion processes, respectively (Natarajan et al., 2003; de Semir et al., 2020). In summary, Galanin modulates tumor progression through multiple pathways, and targeting these pathways may contribute to the development of novel treatments for GBM.

Single-cell sequencing results revealed that the hub genes are widely expressed in GBM tumors and immune cells. LGALS3, which is specifically overexpressed, regulates the cell cycle to promote tumor proliferation, while SERPINA1, which is enriched in immune cells, participates in the remodeling of the tumor microenvironment. These findings confirm that Galanin regulates tumor progression through a dual mechanism: directly promoting cancer cell proliferation and inducing an immunosuppressive microenvironment, thereby influencing patient prognosis via intercellular communication.

The LASSO-Cox prognostic model developed in this study has been validated across multiple cohorts, demonstrating excellent predictive performance and generalizability, and can be used for risk stratification and personalized diagnosis and treatment of GBM patients. Molecular docking studies confirmed that Galanin stably binds to key gene proteins, providing a structural explanation for its oncogenic mechanism. Furthermore, *in vitro* siRNA experiments demonstrated that knocking down CCNA2 and SERPINA1 significantly inhibited GBM cell proliferation, validating their oncogenic roles and providing experimental support for GBM target research.

This study has certain limitations, namely, that the Galanin–CCNA2/SERPINA1 regulatory axis predicted by bioinformatics has not yet been directly validated through experiments involving exogenous ligand intervention. Therefore, follow-up studies will focus on systematic functional validation of this axis. The experimental design will include the following core groups: a solvent control group (ddH_2_O) to rule out solvent interference; a group treated with exogenous Galanin alone to assess the ligand’s baseline inductive effect on target genes; a group treated with a Galanin receptor antagonist alone to observe changes in target gene expression following the blockade of endogenous Galanin signaling; and a group treated with a combination of the antagonist and Galanin to determine whether the antagonist can effectively block the regulatory effect of the exogenous ligand, thereby establishing a direct link between the ligand, receptor, and target gene. Building on this, a pathway-specific inhibitor pretreatment group was added to intervene in potential downstream signaling branches prior to Galanin stimulation, in order to preliminarily identify the major pathways mediating this regulation. Finally, to verify the functional necessity of the target genes, we established a control group treated with siRNA plus solvent, a control group treated with siRNA plus Galanin, a group with co-knockdown of the target genes (CCNA2 and SERPINA1) combined with the solvent control, and a group with target gene knockdown combined with Galanin. By comparing the proliferation and cell cycle phenotypes across these groups, we determined whether knockdown could reverse Galanin’s proliferative effect, thereby fully validating at the functional level the causal chain in which the ligand regulates target gene expression via its receptor and ultimately drives tumor cell proliferation.

## 5 Conclusion

The present study demonstrates that Galanin contributes to glioblastoma progression and constitutes a potential risk factor for this disease. We further identified several key genes—namely CCNA2, BRCA1, SERPINA1, and LGALS3—that are significantly enriched in the PI3K-Akt pathway and exhibit favorable predictive performance in associated prognostic models. *In vitro* experiments showed that knockdown of CCNA2 or SERPINA1 markedly reduced tumor cell proliferation and colony formation. Both genes appear to drive malignant growth in glioblastoma and thus represent viable therapeutic targets. Collectively, these findings provide a theoretical foundation for understanding the mechanistic actions of Galanin and for advancing treatment strategies against glioblastoma.

## Data Availability

The datasets presented in this study can be found in online repositories. The names of the repository/repositories and accession number(s) can be found in the article/Supplementary Material.
