# Supplementary material for: Exploring the causal role and mechanism of galanin in glioblastoma: integration of mendelian randomization, network analysis, molecular docking and experimental validation
Source: Front Pharmacol. 2026 Aug 4;17:1916071. doi: 10.3389/fphar.2026.1916071 (PMC13481792; doi:10.3389/fphar.2026.1916071)

Supplementary Fig. 1: Performing LASSO-COX analysis on 23 intersecting genes using CCGA transcriptomic data

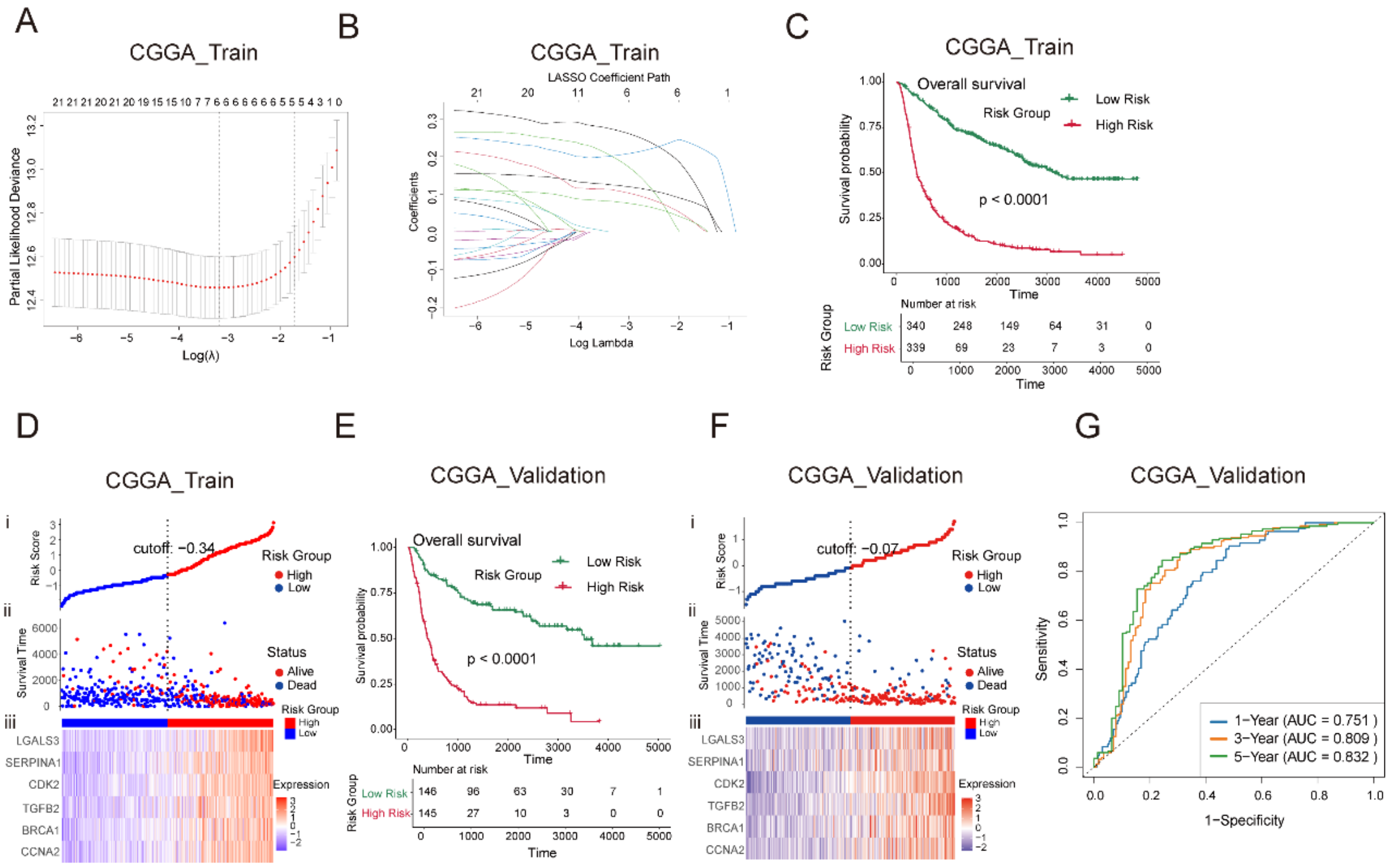

Supplement: Supplementary file 1 [file Image1.pdf]
