# Supplementary material for: Exploring the causal role and mechanism of galanin in glioblastoma: integration of mendelian randomization, network analysis, molecular docking and experimental validation
Source: Front Pharmacol. 2026 Aug 4;17:1916071. doi: 10.3389/fphar.2026.1916071 (PMC13481792; doi:10.3389/fphar.2026.1916071)
Supplement: Supplementary file 2 [file Table2.docx]

Supplementary Table S2: Heterogeneity test of the lVW and MR egger analyses and pleiotropy test (egger intercept)

| Exposure | Outcome | Methods | Cochran's Q | Q-value | p-value (Pleiotropy test) |
| --- | --- | --- | --- | --- | --- |
|  |  |  |  |  |  |
| Galanin peptide levels \|\| id:ebi-a-GCST90012062 | glioblastoma \|\| id:finn-b-C3_GBM_EXALLC | MR Egger | 2.603 | 0.4569 | 0.6437 |
|  |  | Inverse variance weighted | 2.865 | 0.5805 |  |
