# Supplementary material for: Exploring the causal role and mechanism of galanin in glioblastoma: integration of mendelian randomization, network analysis, molecular docking and experimental validation
Source: Front Pharmacol. 2026 Aug 4;17:1916071. doi: 10.3389/fphar.2026.1916071 (PMC13481792; doi:10.3389/fphar.2026.1916071)
Supplement: Supplementary file 3 [file Table4.docx]

Supplementary Table S4: Intersection gene of Galanin peptide, GSE86574 and GSE147352.

| Gene Symbol | Gene Symbol | Gene Symbol | Gene Symbol | Gene Symbol |
| --- | --- | --- | --- | --- |
| PRSS3 | NCS1 | PAK6 | TGFBR2 | TYMS |
| CD4 | MAPK10 | KIF11 | F2RL1 | SSTR2 |
| BRCA1 | B3GAT1 | GLTP | CASP3 | F2R |
| BIRC3 | CCND1 | SSTR1 | ERAP2 | DRD1 |
| RTN4R | CCKBR | EPHA2 | SYK | CASP4 |
| HAGH | BCHE | CCNA2 | HPRT1 | SERPINA1 |
| CA12 | ITGA4 | EGFR | CASP7 | C5AR1 |
| OPRK1 | STAT1 | ITGB1 | CDYL2 | PSMB8 |
| MMP2 | PLAU | THRB | PLK1 | DHFR |
| LYZ | CDK2 | EDNRA | RRM1 | PSMB9 |
| TGFB2 | PCSK2 | MMP14 | DTYMK | DDOST |
| CBX7 | RAC2 | C1R | MAP2K1 | CCL5 |
| PYGL | LGALS3 | AZGP1 | RNASE2 | ITGB8 |
| ERI1 | CTSL | ITGA5 | FABP6 | ITPKA |
| PIK3CG | CASP1 | MAPKAPK2 | ISG20 | ADCY5 |
| CTSS |  |  |  |  |
