# Supplementary material for: Exploring the causal role and mechanism of galanin in glioblastoma: integration of mendelian randomization, network analysis, molecular docking and experimental validation
Source: Front Pharmacol. 2026 Aug 4;17:1916071. doi: 10.3389/fphar.2026.1916071 (PMC13481792; doi:10.3389/fphar.2026.1916071)
Supplement: Supplementary file 4 [file Table5.docx]

| Gene | Degree | Betweenness  Centrality | ClosenessCentrality |
| --- | --- | --- | --- |
| EGFR | 28 | 0.101294977 | 0.594059406 |
| CD4 | 28 | 0.206987779 | 0.618556701 |
| CASP3 | 26 | 0.129302808 | 0.6 |
| CCND1 | 24 | 0.084897209 | 0.576923077 |
| MMP2 | 22 | 0.073258916 | 0.555555556 |
| CCL5 | 20 | 0.071129936 | 0.555555556 |
| STAT1 | 20 | 0.033628715 | 0.545454545 |
| ITGB1 | 18 | 0.020846336 | 0.52173913 |
| CTSS | 18 | 0.051803432 | 0.540540541 |
| CASP1 | 18 | 0.040847374 | 0.526315789 |
| CDK2 | 17 | 0.022484698 | 0.495867769 |
| CCNA2 | 15 | 0.020616671 | 0.491803279 |
| BRCA1 | 13 | 0.071444386 | 0.472440945 |
| PSMB9 | 13 | 0.023944258 | 0.487804878 |
| BIRC3 | 13 | 0.007695867 | 0.483870968 |
| PLAU | 12 | 0.00959717 | 0.487804878 |
| TYMS | 12 | 0.023898342 | 0.458015267 |
| PSMB8 | 12 | 0.02182947 | 0.483870968 |
| LGALS3 | 11 | 0.034281583 | 0.491803279 |
| TGFB2 | 10 | 0.009489807 | 0.483870968 |
| MAP2K1 | 10 | 0.018758729 | 0.454545455 |
| RAC2 | 10 | 0.019815549 | 0.46875 |
| SERPINA1 | 9 | 0.048236351 | 0.465116279 |

Supplementary Table S5: Hub targets screened from PPI network
