# Supplementary material for: Exploring the causal role and mechanism of galanin in glioblastoma: integration of mendelian randomization, network analysis, molecular docking and experimental validation
Source: Front Pharmacol. 2026 Aug 4;17:1916071. doi: 10.3389/fphar.2026.1916071 (PMC13481792; doi:10.3389/fphar.2026.1916071)
Supplement: Supplementary file 5 [file Table7.docx]

Supplementary Table S7: The results of Galanin docked with the hub genes.

| Gene | Docking Score | Confidence Score | Ligand rmsd (Å) |
| --- | --- | --- | --- |
| BRCA1 | -444.52 | 0.9972 | 47.27 |
| CCNA2 | -383.77 | 0.9908 | 35.91 |
| LGALS3 | -342.08 | 0.9790 | 46.39 |
| SERPINA1 | -346.98 | 0.9809 | 25.75 |
